# Case Report: Metachronous oncocytic and colloid carcinoma arising from pancreatic intraductal neoplasms over four decades

**DOI:** 10.3389/fonc.2026.1900534

**Published:** 2026-09-03

**Authors:** Maria Imperatrice, Michael Johannes Pflüger, Mari Mino-Kenudson, Carlos Fernandez-del Castillo, M. Lisa Zhang

**Affiliations:** 1 Department of Surgery, Massachusetts General Hospital, Harvard Medical School, Boston, MA, United States; 2 Department of General and Pancreatic Surgery, The Pancreas Institute, University of Verona Hospital Trust, Verona, Italy; 3 Department of Pathology, Massachusetts General Hospital, Harvard Medical School, Boston, MA, United States

**Keywords:** colloid carcinoma, intraductal neoplasm, intraductal oncocytic papillary neoplasms (IOPN), intraductal papillary mucinous neoplasm (IPMN), oncocytic carcinoma

## Abstract

Intraductal papillary mucinous neoplasms (IPMN) are precursors of pancreatic ductal adenocarcinoma (PDAC) and may progress to distinct invasive phenotypes depending on epithelial subtypes. Tubular carcinoma, often arising from pancreatobiliary epithelium, is clinically aggressive and resembles PDAC, whereas colloid carcinoma, typically derived from intestinal epithelium, has a more indolent course. Intraductal oncocytic papillary neoplasms (IOPN), historically considered a subtype of IPMN, are now recognized as a distinct entity characterized by abundant eosinophilic cytoplasm, complex papillary architecture, and unique molecular alterations. Although the heterogeneity of intraductal pancreatic neoplasms (i.e., IPMN, IOPN, ITPN) is recognized, the mechanisms and temporal dynamics underlying their malignant progression remain poorly understood. We report the case of a female patient in her 80s with a pancreatic body cyst first identified in her 40s. After decades of radiologic stability, she underwent a middle pancreatectomy in 2017, which showed a 1.5 cm oncocytic carcinoma (pT1N0) arising from an IOPN. After surgical resection, she remained disease-free for eight years. In 2025, she developed a new pancreatic head cyst and underwent pancreaticoduodenectomy, which revealed a colloid carcinoma (pT3N0) arising from an intestinal-type IPMN. Molecular profiling of the 2025 specimen demonstrated shared driver alterations in the intestinal-type IPMN and associated colloid carcinoma (*KRAS* p.G12V and *GNAS* p.R201C), with *BRAF* p.D594Y detected in the IPMN component and MTAP deletion detected in the invasive component. Sequencing of the 2017 specimen was not feasible due to limitations of archival tissue. This case describes the metachronous clinical presentation and histologic diagnosis of oncocytic and colloid carcinomas eight years apart within a clinical history spanning nearly four decades. Because sequencing of the 2017 tumor was unsuccessful, clonal independence cannot be established. The case supports prolonged surveillance and further molecular study.

## Introduction

Intraductal papillary mucinous neoplasms (IPMN) are precursors of pancreatic ductal adenocarcinoma (PDAC) and may progress into distinct subtypes of invasive carcinoma (1–3). At the molecular level, IPMNs commonly harbor early activating events in MAPK and G-protein signaling pathway genes (most notably *KRAS* and *GNAS*), whereas additional alterations in tumor suppressor and mTOR-pathway genes are enriched in high-grade dysplasia and invasive carcinoma, supporting a progression model with sequential mutations in driver genes (3–5). The phenotype of the invasive component is strongly associated with the epithelial subtype in the preceding IPMN (6). Invasive tubular carcinoma mostly arises from pancreatobiliary-type epithelium but may also originate from gastric-type or intestinal-type IPMNs. Invasive tubular carcinoma arising in IPMN is associated with a more aggressive course resembling conventional PDAC (7). In contrast, invasive colloid carcinoma is less frequently encountered and derived almost exclusively from intestinal-type IPMN. It is associated with a more favorable prognosis due to its more well-circumscribed growth pattern (with tumor cells floating in pools of mucin) and lower frequency of lymph node metastases (3, 8–12). While the heterogeneity of invasive IPMN subtypes is recognized, the biological mechanisms and temporal dynamics underlying their progression remain poorly understood (13). Patients with IPMN also have an increased risk of developing a concomitant PDAC, unrelated to the IPMN itself, and therefore require long-term surveillance (3, 13, 14).

Alongside these conventional pathways, intraductal oncocytic papillary neoplasms (IOPN) represent an uncommon oncocytic lineage within the spectrum of intraductal pancreatic neoplasms, characterized by complex papillae and oncocytic cells; when invasive, they give rise to oncocytic carcinoma (15). IOPNs are now recognized and classified as distinct from conventional IPMN in contemporary classification and display a unique molecular profile, most notably recurrent *PRKACA/PRKACB* fusions and typically absent *KRAS/GNAS* mutations, with favorable reported clinical outcomes more similar to that of colloid carcinoma than tubular carcinoma (16–19).

This case report describes metachronous clinical presentation and histologic diagnosis of oncocytic and colloid carcinomas eight years apart. This rare clinical scenario highlights histologic heterogeneity within the spectrum of intraductal neoplasms and supports continued, individualized surveillance after curative resection.

## Case presentation

### Initial presentation and first resection (2017)

**Figure 1:**
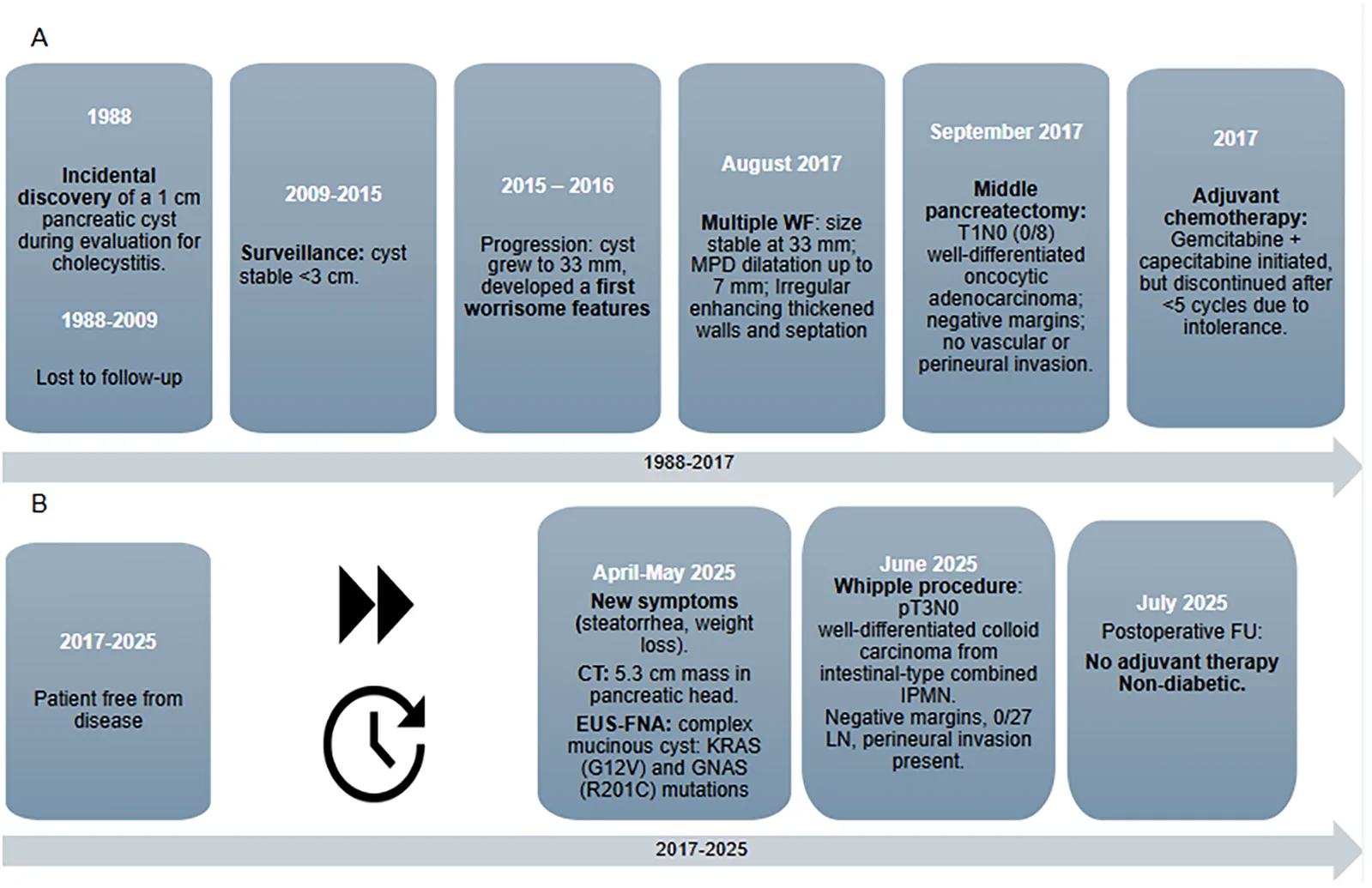
Clinical timeline of disease progression. **(A)** 1988–2017: initial detection, follow-up, and treatment of first pancreatic cyst, which was revealed to be an intraductal oncocytic papillary neoplasm (IOPN) with invasive oncocytic carcinoma. **(B)** 2017- 2025: post-initial resection time course and development of second malignancy (intestinal-type IPMN with invasive colloid carcinoma), treated with Whipple resection without adjuvant therapy.

**Figure 2:**
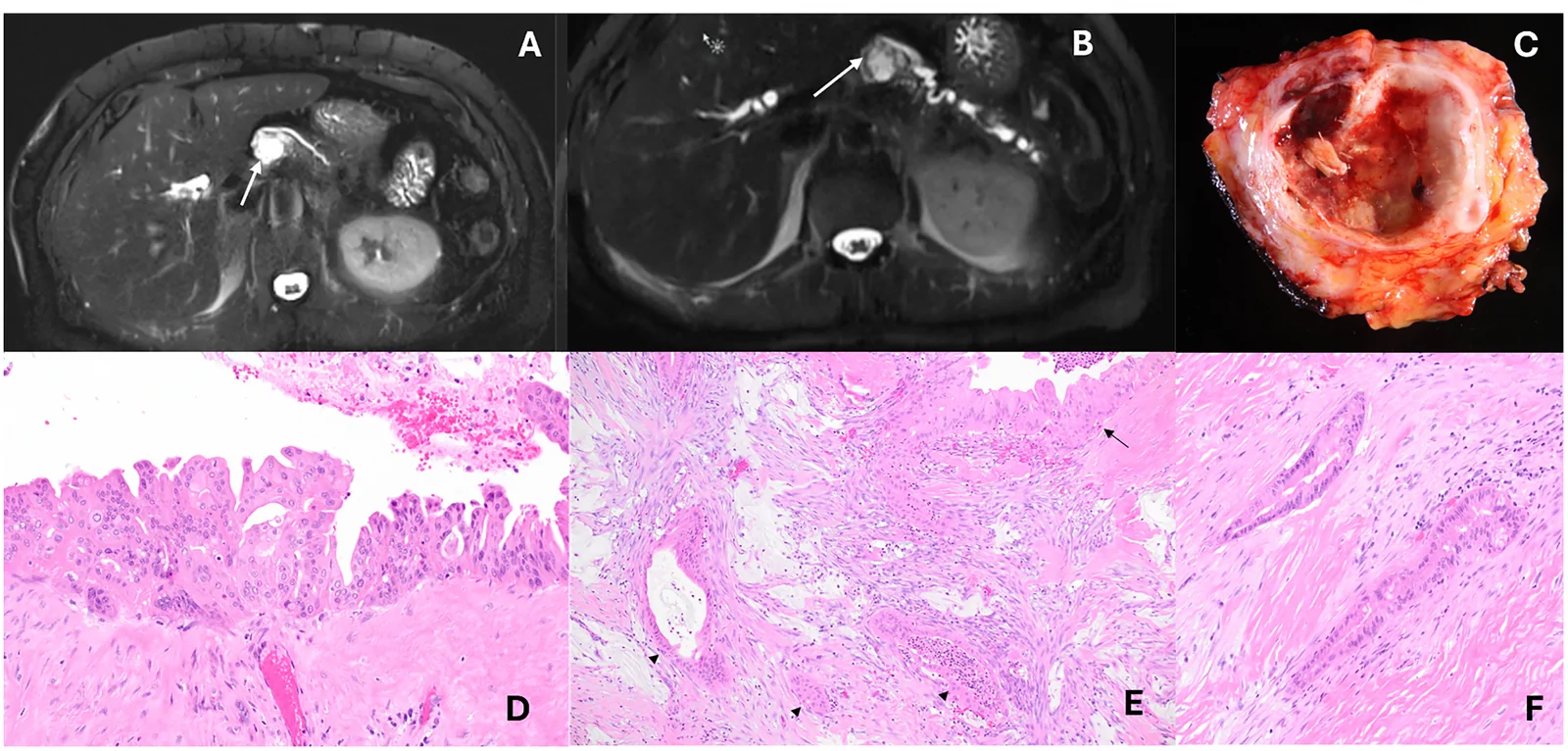
Radiology and pathology of the initial cystic lesion. **(A)** MRI from 2015 showing a cystic lesion without high-risk and one worrisome feature (33 mm). **(B)** Follow-up MRI demonstrating development of irregular enhancing septations, thickened walls in the superior pole, and main pancreatic duct dilatation up to 7 mm. **(C)** Gross photograph of the middle pancreatectomy specimen. **(D)** Histological image of the intraductal component with predominantly oncocytic epithelium showing complex architecture. The lining epithelial cells on the right side contain slightly less cytoplasm, imparting a more pancreatobiliary-type appearance (H&E, 200X). **(E)** Intraductal oncocytic papillary neoplasm (arrow) with underlying rupture and oncocytic invasive carcinoma glands (arrowheads) (H&E, 100X). **(F)** Angulated invasive carcinoma glands further from area of rupture (H&E, 200X).

A woman in her 40s was found to have a small 1.0 cm pancreatic body cyst incidentally in 1988 (**Figure 1A**). She was lost to follow-up until 2009, when the cyst was again visualized. The indication for the 2009 imaging and details of surveillance before referral to our institution in 2015 were unavailable, though at the initial 2015 visit, the cyst was described to have been stable without worrisome features. From 2015, MRI was performed approximately every 6 months and later annually, showing growth to 3.3 cm in 2015 (**Figure 2A**). By 2017, MRI demonstrated the emergence of multiple worrisome features, including progressive main pancreatic duct dilation of up to 7 mm with irregular septations and wall thickening, raising suspicion for IPMN with malignant transformation (**Figure 2B**). Serum tumor markers (CEA, CA 19-9) remained within normal limits. Because she was healthy with few comorbidities, a middle pancreatectomy was planned to preserve pancreatic function and reduce the risk of postoperative diabetes, with possible conversion to pancreaticoduodenectomy or distal pancreatectomy based on the intraoperative findings.

Grossly, a 3.0 × 2.7 × 2.0 cm cystic mass with abundant gelatinous material was resected (**Figure 2C**). Histopathologically, the lesion was diagnosed as a 1.5 cm IOPN (previously oncocytic IPMN at the time of original diagnosis) with complex papillae composed of oncocytic cells, mixed with focal pancreatobiliary-type areas (**Figure 2D**). The invasive component showed abundant mucin pools, some lined by oncocytic epithelium; however, many were associated with gland rupture, inflammation, and giant cells, consistent with a colloid-like appearance secondary to rupture rather than true colloid carcinoma (**Figures 2E, F**). The tumor was graded as well-differentiated (G1) and staged as pT1N0 invasive carcinoma (now pT1c under AJCC 8th edition staging criteria). There was no perineural invasion, vascular invasion, or lymph node metastasis (0/8); resection margins were negative. Despite favorable pathologic features, adjuvant gemcitabine-capecitabine was recommended based on available data at the time; treatment was stopped after four cycles because of intolerance. CT at approximately 6-month intervals during the first postoperative year showed no recurrence or metastasis. Routine imaging was then discontinued; CT/MRI in 2021 was stable, and the next CT was obtained for symptoms in 2025.

### Second presentation and resection (2025)

**Figure 3:**
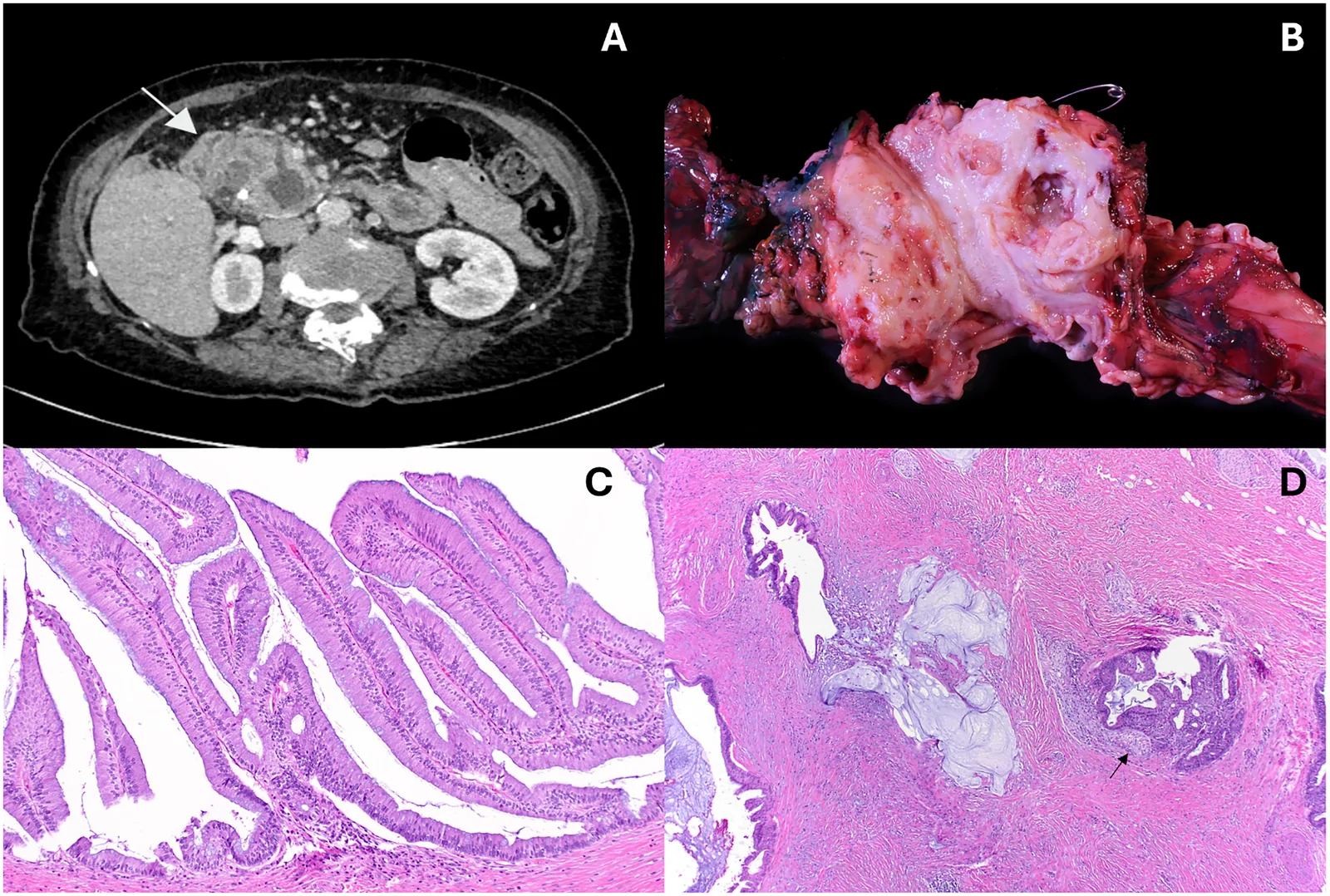
Radiology and pathology of the second cystic lesion. **(A)** CT imaging of the metachronous cyst before the second surgery (arrow). **(B)** Gross photograph of the pancreaticoduodenectomy specimen. **(C)** Histological image of the intraductal papillary component showing long villiform architecture, consistent with intestinal-type epithelium (H&E, 200X). **(D)** Invasive colloid carcinoma with large duct-pattern invasion and perineural invasion (arrow) (H&E, 40X).

In 2025, the patient presented with new symptoms including steatorrhea, pale stools, epigastric discomfort, and a 10-pound weight loss over the preceding two months (**Figure 1B**). CT imaging showed a 5.3 cm cystic mass in the pancreatic head, suspicious for IPMN with malignant transformation (**Figure 3A**). Laboratory workup showed elevated serum alkaline phosphatase and ALT, but normal CEA and CA 19-9. EUS-FNA and ERCP revealed a complex cystic lesion with atypical mucinous epithelium and a malignant-appearing biliary stricture, prompting the placement of a biliary stent. Molecular analysis of the pancreatic cyst fluid identified *KRAS* G12V and *GNAS* R201C mutations, confirming a neoplastic mucinous cyst. Pancreatic enzyme replacement therapy was prescribed for exocrine insufficiency.

After multidisciplinary discussion, the patient underwent a pancreaticoduodenectomy in 2025 (**Figure 3B**). Final pathology demonstrated a well-differentiated invasive colloid carcinoma with large duct-pattern invasion (pT3N0) arising from an intestinal-type IPMN (**Figures 3C, D**). Perineural invasion was present, but no vascular invasion was identified. There were no lymph node metastases (0/27), and resection margins were negative. The postoperative course was complicated by gastric outlet obstruction requiring readmission. Further diagnostic work-up with EUS showed narrowing of the gastrojejunostomy, which was treated successfully with stenting and nasojejunal feeding. The patient was discharged after resuming stable oral intake. Given her age, her prior intolerance to chemotherapy, and the favorable pathology, adjuvant treatment was not recommended.

## Molecular testing results

Given the unusual metachronous occurrence of histologically distinct intraductal neoplasm-associated carcinomas, molecular testing was pursued. Poor archival tissue quality precluded successful sequencing of the 2017 specimen despite attempts at both academic (Mass General Brigham) and commercial (Caris Life Sciences) laboratories. Therefore, a clonal relationship between the 2017 and 2025 tumors could neither be established nor excluded. Comprehensive profiling of the IPMN and its corresponding invasive carcinoma from the 2025 specimen was performed by Caris Life Sciences using hybrid-capture next-generation sequencing on an Illumina NovaSeq 6000 platform capturing over 700 clinically relevant genes plus over 20,000 additional genes. A customized bioinformatics pipeline to detect sequence variants/indels, copy-number alterations, and genomic signatures, along with whole-transcriptome RNA sequencing for variant transcript and fusion detection was used. Shared alterations in both the IPMN and invasive carcinoma included *KRAS* p.G12V and *GNAS* p.R201C. In addition, the IPMN harbored a *BRAF* p.D594Y mutation, while the carcinoma/invasive component showed MTAP deletion. Tumor mutation burden was low (3 mutations/Mb), and no actionable gene fusions were detected. MTAP immunohistochemistry (Santa Cruz, clone sc-100782, 1:100) showed retained cytoplasmic expression in the 2017 carcinoma and loss of cytoplasmic expression in the 2025 carcinoma.

## Discussion

IPMNs are precursors of highly lethal PDAC, and their management remains a clinical challenge due to the variable risk of malignant transformation (13, 20). This case represents a rare yet informative clinical observation in which two histologically distinct neoplastic cysts with associated invasive carcinomas were diagnosed over a clinical history spanning nearly four decades.

This unusual clinical trajectory underscores several critical points in the natural history and biology of intraductal pancreatic neoplasms and their associated carcinomas. First, the exceptionally long clinical history in this case, which spans almost 40 years from initial cyst detection to diagnosis of the second invasive carcinoma, reinforces the slow growth yet unpredictable and insidious behavior of intraductal pancreatic neoplasms. Although current management guidelines recommend resection based on defined radiographic high-risk stigmata or worrisome imaging features (13), emerging long-term data suggest that stability over time does not equate to biological quiescence. Multiple large-scale observational studies have demonstrated that a subset of patients may progress to malignancy after five or more years of apparent cyst stability, even those without worrisome features (21, 22). Importantly, data from long-term surveillance cohorts show that the risk of pancreatic cancer remains significantly elevated compared to the general population, particularly in younger patients and in those with cysts ≥1.5 cm in diameter (23, 24). Therefore, the potential for malignant transformation occurring relatively late reinforces the need for individualized, extended follow-up strategies that span well beyond five years, particularly in patients with favorable performance status and prolonged life expectancy who would be fit to undergo re-resection.

The last stable CT/MRI was in 2021, about four years before symptoms developed, so the timing and growth of the second lesion cannot be reconstructed. Intermittent surveillance therefore did not capture when the new lesion developed. Its combination of pT3 disease and perineural invasion with well-differentiated, node-negative histology falls within the range reported in a recent 50-case colloid carcinoma series (pT3, 35%; node-negative, 80%; perineural invasion, 28%), which also showed longer median survival than tubular carcinoma (69 versus 31 months) (12).

The histologically distinct second neoplasm is compatible with multifocal disease and raises the possibility of a pancreatic “field effect.” However, without sequencing of the 2017 tumor, clonal independence and a field effect cannot be established; a clonal relationship or chance occurrence remains possible (3, 10).

Historically, oncocytic lesions were classified as one of the four IPMN subtypes (14). However, accumulating morphological and molecular evidence and particularly the absence of *KRAS/GNAS* mutations and the presence of *PRKACA/PRKACB* fusions, led to their reclassification from oncocytic-type IPMN to IOPN, a distinct entity in the WHO 2019 classification (15–17, 20). Although IOPNs are now recognized as a distinct entity, these lesions almost always have admixed gastric, intestinal, and/or pancreatobiliary areas (17, 25). This heterogeneity raises the question whether IOPNs may still exist within the spectrum of mixed-type IPMN rather than a completely independent lineage a perspective that remains debated (25).

From a molecular standpoint, IPMNs are most commonly characterized by early activating events in the MAPK pathway and G-protein signaling, particularly *KRAS* and *GNAS*. The *GNAS* alterations are largely restricted to IPMNs (especially enriched in intestinal-type IPMNs) and are generally absent in IOPNs (5, 26). The shared *KRAS* p.G12V and *GNAS* p.R201C mutations in both this patient’s 2025 intestinal-type IPMN and the associated colloid carcinoma support an IPMN-related mucinous lineage and are consistent with the well-established role of *KRAS/GNAS* as common early driver events in IPMN. The patient’s IPMN component also harbored *BRAF* p.D594Y, a non-V600E mutation, that is typically RAS-dependent and supports MAPK-pathway activation.

MTAP deletion is a recurrent event in PDAC, reported in approximately 20-30% of cases. It is closely linked to 9p21 loss with frequent co-deletion of *CDKN2A* in pancreatic cancer (27, 28). Moreover, the presence of MTAP deletion confined to the invasive carcinoma component of this patient’s more recent lesion is compatible with additional genomic diversification during progression. MTAP immunohistochemistry is a surrogate marker for genetic MTAP loss. The findings of retained cytoplasmic expression in the 2017 lesion but cytoplasmic loss in the 2025 lesion, however, suggest that MTAP was not deleted in the original carcinoma.

Our study has several limitations. Sequencing of the 2017 tumor was unsuccessful, preventing assessment of the clonal relationship; MTAP immunohistochemistry cannot substitute for comparative sequencing. A pancreatic field effect therefore cannot be established, and chance occurrence remains possible. Clinical germline testing was not performed, so an inherited predisposition cannot be excluded. As a single-patient case report, the study cannot provide incidence estimates or establish causal relationships regarding metachronous intraductal neoplasm-associated carcinomas. The long clinical interval also introduces variability because of evolving diagnostic criteria and imaging/reporting standards. Preoperative cyst risk stratification (e.g., imaging/EUS/cyst fluid/cytology) may miss focal high-grade dysplasia or early invasion (4, 7, 29). Finally, the lack of systematic multi-regional sampling and the scope of the testing platforms limit assessment of intralesional heterogeneity and the full genomic landscape.

## Conclusion

This case report describes the metachronous clinical presentation and histologic diagnosis of oncocytic and colloid carcinomas eight years apart within a nearly four-decade clinical history. It highlights not only the heterogeneity of these intraductal pancreatic lesions and their invasive phenotypes but also illustrates the gaps in our understanding of disease progression. Although the findings suggest heterogeneity and multifocality, the absence of sequencing data from the first tumor precludes establishing clonal independence or divergent molecular progression. Nevertheless, this case underscores the need for long-term surveillance strategies and deeper biological insights particularly into the mechanisms that drive late malignant transformation in patients with intraductal pancreatic neoplasms.

## Data Availability

All relevant data is contained within the article: The original contributions presented in the study are included in the article. Further inquiries can be directed to the corresponding author.
